# Reduced diversity of gut microbiota in two *Aedes* mosquitoes species in areas of recent invasion

**DOI:** 10.1038/s41598-018-34640-z

**Published:** 2018-10-31

**Authors:** Fausta Rosso, Valentina Tagliapietra, Davide Albanese, Massimo Pindo, Frédéric Baldacchino, Daniele Arnoldi, Claudio Donati, Annapaola Rizzoli

**Affiliations:** Fondazione Edmund Mach, Research and Innovation Centre, San Michele all’Adige, Trento, Italy

## Abstract

*Aedes* mosquitoes are considered highly successful global invasive species and vectors of several pathogens of relevance for public health. Their midgut’s microbiota can play an important role in affecting not only their vectorial competence but also their fitness, physiology, food digestion, metabolism, immunity and adaptation to new environmental conditions. Using high-throughput sequencing we compared the microbiota of *Aedes albopictus* collected in Italy with those reported in populations from France and Vietnam. We also analysed *Aedes koreicus* gut microbiota for the first time. We found remarkable individual difference along with common bacterial taxa in both species. *Ae. albopictus* collected in Italy had a lower richness and a different composition of microbiota in respect to specimens collected in France and Vietnam. It also showed a core microbiota formed mainly of bacteria of the genus *Pseudomonas*. Overall, the two *Aedes* species (*Ae*. *albopictus* and *Ae*. *koreicus*) collected in Italy, showed a large core microbiota with 75.98% of the identified Operational Taxonomic Units. Furthermore, *Ae*. *albopictus* had 2.5% prevalence of *Wolbachia* and 0.07% of *Asaia* spp, while *Ae*. *koreicus* had 14.42% of *Asaia* spp. and no *Wolbachia*. This study provides new informations on the spatial variation of the midgut bacterial communities in mosquitoes of medical relevance within areas of recent invasion and provide the basis for further studies aimed at assessing the effects of such variation on vectorial capacity for a range of pathogens.

## Introduction

Biological invasion of alien species represents a rising global issue. In fact, invasive species often exert a negative impact on local biodiversity and economy, including the impact on human and animal health as consequence of pathogen transmission^1,2^.

The mosquito species *Aedes (Stegomyia) albopictus* (Skuse, 1894) and *Aedes (Finlaya) koreicus* (Edwards, 1917) are native to South East Asia and are now invading several European countries posing an increasing threat to human and animal public health^3^. *Ae*. *albopictus*, considered as one of the top 100 most invasive species of the world^4^, is a competent vector for at least 26 arboviruses^5^, including dengue, chikungunya and Zika, and it was first detected in Europe in 1979^6^ and in the Province of Trento (Italy) in 1996^7^. *Ae*. *koreicus*, recorded in Italy for the first time in 2011^8^ transmits the parasitic nematode *Dirofilaria immitis*^9^, has been proven to transmit experimentally the Japanese Encephalitis virus^10^, while the competence to transmit viruses such as dengue, chikungunya and Zika requires experimental validation.

It should be taken into consideration that colonization of new habitats often represents a challenge for the invasive species itself, since the adaptation to the new environment is highly costly due to the variety of new selective pressures encountered by the introduced species^11^.

The structure of the gut microbiota is a sensitive indicator of health status in humans and animals, where a high microbiota diversity is usually associated with healthy conditions of the host^12^. Recently, it has been shown that environmental disturbances can also cause a loss of diversity in the gut microbiota of wild species^13^, leading to the hypothesis that gut microbiota diversity can be used as a measure of fitness of a wild species invading a new habitat. In case of arthropods, the role of microbial communities inhabiting gut are increasingly under study. As in mammals, microbiota takes part in the characteristic physiology of the species and plays a key role in food digestion, metabolism, immunity and adaptation to new environmental conditions^14,15^, even if most insect guts generally contain few microbial species^14^. When the invasive species is an arthropod vector of pathogens, testing its vectorial capacity and ability to spread into new environment is essential in term of actual disease risk estimate. Infact, in blood-feeding insect vectors microbiota plays another important function, namely it affects vectorial competence, i.e. their ability to transmit pathogens to their target species. Indeed, gut microbiota modulates actively the proliferation and development of pathogens, and any pathogen entering the host must interact both with the midgut and the resident bacteria^16,17^. All mosquito-transmitted pathogens complete their life-cycle in the gut where they are exposed to barriers and immunity mediators activated by the microbiota^17,18^.

Recent studies have highlighted the ability of insect-associated bacteria to modulate vector competence for arboviruses and other pathogens through mechanisms such as immune response activation, resource competition, or production of anti-viral molecules^17^. Therefore, monitoring the gut microbiota diversity during colonization of new environments can represent a novel approach in terms of evaluation of the vectorial capacity and the actual disease hazard estimate, combining indications on potential vector competence and fitness. Vectorial capacity is in fact defined as the capability for disease transmission by a vector to a host, as influenced by behavioral, ecological and environmental factors, such as population density, host preference, feeding habits or frequency, duration of latent period, or longevity.

The development of metataxonomic analysis based on High Throughput Sequencing (HTS) has made possible to detect a deeper level of microbial diversity in animal hosts and recent studies demonstrated that this technique can be successfully applied to mosquito’s gut^19,20^. Since the environment (and the adaptation to it) is known to influence the association between host and microbiota community composition in mosquitoes^19,21,22^, we hypothesized that gut microbiota from introduced populations should be similar to each other, but different to autochthonous populations, at least in terms of richness. To assess this assumption we compared the midgut microbiota from *Ae*. *albopictus* samples collected in northern Italy with data recently published on introduced populations from France and autochthonous populations from Vietnam^20^. Furthermore, we analyzed and compared wild-caught samples of *Aedes* species (*Aedes albopictus* and *Aedes koreicus*) collected in the same region, in order to compare their microbial community and assess potential hazards of pathogens transmission in our territory. Finally this is the first study reporting data on the gut microbiota of *Ae*. *koreicus*.

## Results

Using publicly available data, we compared the gut microbiota of a population of *Ae*. *albopictus* from the Trentino region, an area located in northern Italy, to French samples of the same species, and to samples from Vietnam, that belongs to the area endemically occupied by this species, before its recent global spread. We also characterized the structure, composition and diversity of the gut microbiota of *Ae*. *albopictus* and *Ae*. *koreicus* from the study area.

### Comparative analysis of Italian, French and Vietnamese *Ae*. *albopictus* microbiota

**Figure 1:**
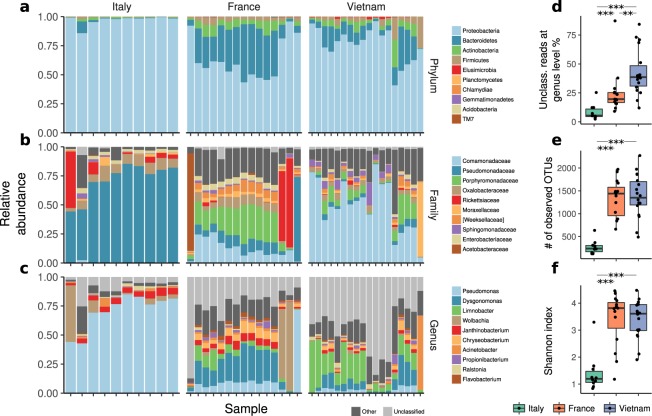
Taxonomical composition of Italian, French and Vietnamese *Ae*. *albopictus* microbiota (**a**) by Phylum, (**b**) by family, (**c**) by genus. Box-plot of the % of unclassified reads at genus level per country (**d**); numbers of observed OTUs per country (**e**) and Shannon index per country (**f**). **p < 0.05, ***p < 0.01.

The taxonomic structure at the Phylum, Family and Genus levels showed clear diversification between the Italian, French and Vietnamese populations (Fig. 1a–c and Supplementary Tables S1–S3).

At the Phylum level, the composition of midgut bacterial community in the Italian population was almost exclusively composed by *Proteobacteria* (mean 97%, sd 4.1%, Supplementary Table S1), while the French and Vietnamese populations, although still containing a high proportion of *Proteobacteria* (mean 62.7% sd 17.5% and mean 76.1% sd 14.7%, respectively, Supplementary Table S1), also contained variable fractions of *Bacteroidetes* (France mean 27.7%, sd 15.2%, Vietnam mean 12.7%, sd 10%, Italy mean 1.5% sd 3%, Supplementary Table S1), *Actinobacteria* (France mean 6.6%, sd 3.3%, Vietnam mean 5.3%, sd 5.3%, Italy mean 0.9% sd 0.7%, Supplementary Table S1), *Firmicutes* (France mean 3%, sd 1,6%, Vietnam mean 5%, sd 7%, Italy mean 0.4% sd 0.4%, Supplementary Table S1) and other representatives with negligible quantity. These differences were confirmed at the Family and Genus level (Supplementary Tables S2 and S3). While the Italian population was dominated by the Family *Pseudomonadaceae* (71%, sd 15%, Supplementary Table S2), Genus *Pseudomonas* (71%, sd 15%, Supplementary Table S3), the French and Vietnamese populations showed a much more complex structure, with high prevalence of bacteria from the family *Comamodanaceae* (12%, sd 7.8%, and 49.2%, sd 22.3%, respectively, Supplementary Table S2), in particular from the genus *Dysgonomonas* for the French samples (18.2%, sd 11%, Supplementary Table S3) and *Limnobacter* for the Vietnamese samples (17.4%, sd 13.2%, Supplementary Table S3). This family was almost absent from the Italian samples (see Fig. 1b,c and Supplementary Table S2).

The simplified microbiota structure in the Italian population was also confirmed by considering the number of unclassified reads (Fig. 1d), which is a measure of the fraction of the microbiota that is composed by organisms that are not present in the reference taxonomic databases. This fraction was much higher in the French and Vietnamese populations, suggesting the presence of a large fraction of not yet characterized organisms, compared to the Italian population. In particular, we found statistically significant differences in terms of unclassified reads at genus level (Fig. 1d) between Italy and France (Wilcoxon rank sum test, FDR corrected P-value = 6 × 10^−4^), Italy and Vietnam (P = 8.7 × 10^−6^) and France and Vietnam (P = 10^−3^). In addition, the Italian population showed a significantly reduced α-diversity when compared to France and Vietnam both in terms of number of observed OTUs (Fig. 1e, P = 9.2 × 10^−7^ for both France and Vietnam) and of Shannon entropy (Fig. 1f, P = 6.1 × 10^−5^ for France and P = 2.1 × 10^−5^ for Vietnam), while France and Vietnam were not statistically different (Fig. 1e, P = 0.87, and Fig. 1f, P = 0.58, respectively; Supplementary Table S4).

**Figure 2:**
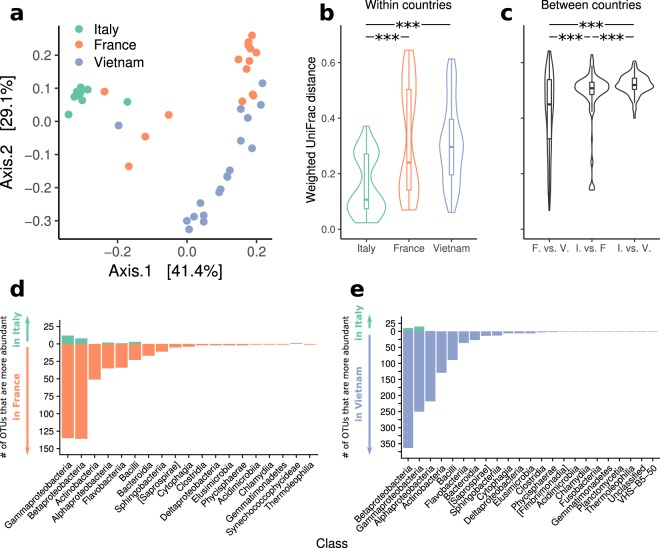
(**a**) Principal coordinates analyses (PCoA) of weighted UniFrac distances, (**b**) within countries distances (**c**) between countries distances. Number of OTUs that are significantly more abundant in (**d**) France or Italy and (**e**) Vietnam or Italy, grouped by class (P < 0.01 DESeq’s Wald significance test, Benjamini & Hochberg FDR correction).

To further investigate if the three different populations corresponded to clearly differentiated structure of the gut microbiota of *Ae*. *albopictus*, we computed the $\beta$-diversity of the samples using the unweighted Unifrac, weighted Unifrac and Bray-Curtis dissimilarity. A plot of the first two axes of the PCoA of the distances amongst the samples calculated using the weighted UniFrac distance showed three distinct clusters, corresponding to the Italian, French and Vietnamese samples (Fig. 2a).

This result is confirmed by the PCoA plots using the unweighted UniFrac distance and the Bray-Curtis dissimilarity (see Supplementary Figures S1 and S2). A permutational multivariate analysis of variance using distances (PERMANOVA) showed that the three different populations (Italian, French and Vietnamese) were significantly different (P < 10^−4^ for the unweighted, weighted UniFrac distances and for Bray-Curtis dissimilarity, see also Fig. 2c). To confirm that the Italian population was clearly distinct from and less diverse than the French and Vietnamese ones, we compared the distributions of the distances within and between the three populations (Fig. 2b,c). Variability within Italy and France and within Italy and Vietnam was significantly different (P = 7.6 × 10^−7^ and P = 3.3 × 10^−10^ respectively, see Fig. 2b), but not within France and Vietnam (P = 0.96). Similarly, distances between countries highlighted the closeness between France and Vietnam, compared to Italy and France and Italy and Vietnam (P = 1.4 × 10^−5^ P = 7.6 × 10^−13^ respectively, Fig. 2c). We then examined which were the taxa differently distributed between Italy and France and Italy and Vietnam. As expected, we found that in most cases the difference between the Italian population and the others was due to taxa significantly more abundant in France and Vietnam than in Italy (P < 0.01, Wald significance test, Benjamini & Hochberg FDR correction, Fig. 2d,e for a summary at the class level, and Supplementary Tables S5 and S6). However, in *Gamma-* and *Betaproteobacteria* only a few OTUs were significantly more abundant in the Italian population, suggesting that the structure of the microbiota of these samples is the result of a general rearrangement of the relative abundances of the different species, rather than being just a simplified version of the French or Vietnamese ones.

### Midgut microbiota of *Ae*. *albopictus* vs. *Ae*. *koreicus*

**Figure 3:**
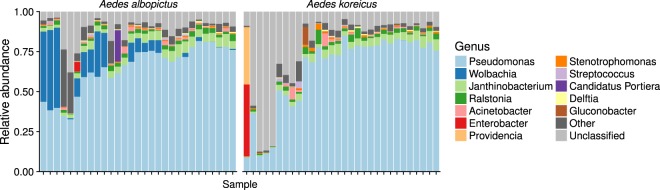
Taxonomical composition of midgut microbiota of *Ae*. *albopictus* and *Ae*. *koreicus* at genus level (Supplementary Table S9).

*Ae*. *koreicus* is a species very recently introduced in Italy. To investigate if stress factors due to the adaptation to an alien environment (which appears to be very strong in *Ae*. *albopictus*) dominated over the genetic differentiation of these two species in determining the structure of the midgut microbiota, we compared samples from the Italian populations of *Ae*. *albopictus* and *Ae*. *koreicus*. After rarefaction (without replacement) 2 153 OTUs were clustered into 9 Families and 12 Genus (Supplementary Tables S7–S9). Despite extensive variation between individuals of the same host species in the composition of their gut microbiota (Fig. 3), the comparison between *Ae*. *albopictus* and *Ae*. *koreicus* revealed a large shared core (75.98% of the reads), complemented by a smaller fraction specific to each of the two species. At Phylum level *Proteobacteria* and *Firmicutes* dominated the community (Supplementary Table S7). At Family level *Pseudomonadaceae* was the most abundant so far, followed by *Rickettsiaceae*, *Oxalobacteraceae*, *Moraxellaceae*, *Enterobacteraceae*, *Xanthomonadaceae*, *Halomonadaceae*, *Acetobacteraceae* and *Comamodanaceae* (Supplementary Table S8).

**Figure 4:**
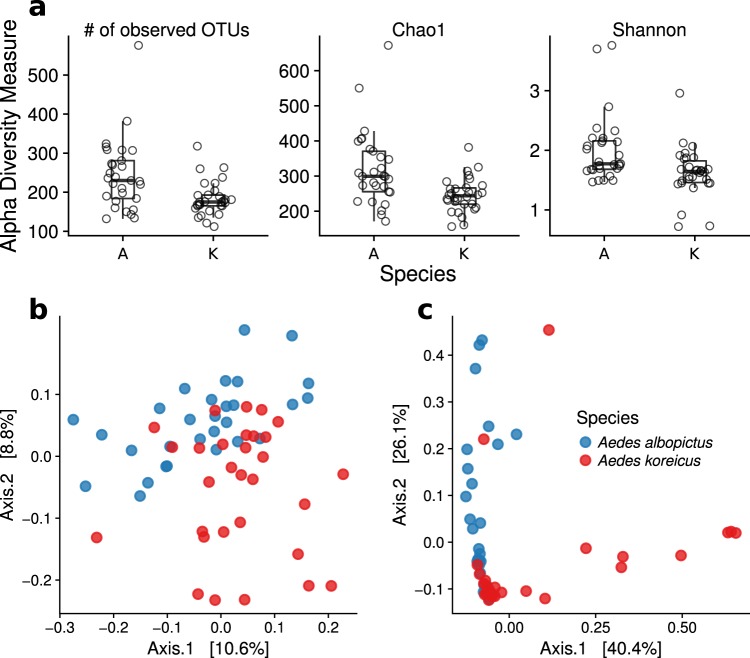
Comparison of α- and β-diversity for *Ae*. *albopictus* (A) and *Ae*. *koreicus* (K). (**a**) Observed number of OTUs, Chao1 estimator and Shannon entropy. In all cases the difference was statistically significant (P = 0.0027, 8.4 × 10^−4^ and 9.5 × 10^−4^ for number of OTUs, Chao1 and Shannon entropy, respectively, Wilcoxon rank-sum test). (**b**) Principal coordinates analyses (PCoA) of unweighted (**b**) and weighted (**c**) UniFrac distances.

The number of observed OTUs, the Chao1 estimator and Shannon entropy indicated a significantly higher α-diversity in *Ae*. *albopictus* compared to *Ae*. *koreicus* (Fig. 4a and Supplementary Table S10, P = 0.0027, 8.4 × 10^−4^ and 9.5 × 10^−4^ for number of OTUs, Chao1 and Shannon entropy, respectively, Wilcoxon rank-sum test, uncorrected p-values). Moreover, beside their different richness, the samples from the two species had a significantly different gut microbiota as resulted from a PERMANOVA test (9 999 permutations) on the β-diversity (P < 10^−4^, P < 10^−4^ and P = 9 × 10^−4^ for unweighted UniFrac and weighted UniFrac, respectively, Fig. 4b,c).

The core microbiota was largely composed by species of the genus *Pseudomonas* that alone accounted for an average of 64.63% and 63.38% of the reads in *Ae. albopictus* and in *Ae*. *koreicus*, respectively (core OTUs are listed in Supplementary Table S11). In 7 samples of *Ae*. *koreicus* there was a large contribution (up to the 14.42%) from an unidentified genus of the family *Acetobacteraceae* (see Supplementary Table S8). According to BLAST, the closest relative genus belonged to *Asaia* (97% percentual identity). We found that only 3 OTUs were specific to *Ae*. *albopictus*, corresponding to 9.81% of the total *Ae*. *albopictus* abundance (OTUs listed in Supplementary Table S12), while no OTUs were specific to *Ae. koreicus*. Using indicator species analysis we confirmed the three OTUs specific to *Ae*. *albopictus*, additionally identifying 8 OTUs of Phylum *Proteobacteria* specific to *Ae*. *albopictus*, and three specific to *Ae*. *koreicus* (Supplementary Table S13).

Two out of this three *Ae. albopictus*-specific OTUs were classified as belonging to the genus *Wolbachia* which resulted to be the dominant component in three samples from this species. No OTUs classified as *Wolbachia* were found in *Ae. koreicus*. In order to characterize more precisely the differences in the taxonomic composition of the samples from the two species, we identified all those OTUs with significant different relative abundance (Wilcoxon rank-sum test P < 0.05, FDR corrected). In addition to the species-specific OTUs identified above, we obtained 7 relatively rare OTUs with relative abundances ranging from a few percent of the total microbiota to less than 10^−3^ (Supplementary Table S14).

## Discussion

Invasion by alien exotic mosquito species, such as *Ae. albopictus* and *Ae. koreicus*, is now occurring in an increasing number of european countries (see https://ecdc.europa.eu/en/disease-vectors/surveillance-and-disease-data/mosquito-maps). These processes therefore enhance the probability of new diseases outbreak events within new areas. Therefore the needs is now to improve scientific knowledge on all the factors affecting mosquitoes vectorial capacity under a “pathobiome” approach^23^. Northern Italy is the only site in Europe where both *Ae*. *albopictus* and *Ae*. *koreicus* are considered established. Therefore, we took this unique opportunity to compare their gut microbiota not only for the potential health hazard they represent, but also because of the lack of knowledge on the midgut microbiota composition of *Ae*. *koreicus*. This latter species, although introduced in northern Italy few years ago^8^, is well established in an area of 3000 km^2^ and its spreading is still ongoing^24^.

The mosquito’s gut microbiota composition varies as a result of complex interactions between environmental characteristics and developmental stages. Nonetheless the common characteristics of the habitat of *Aedes* spp. coupled with the high inter-individual diversity, makes it difficult to determine the geographic origin based on microbiota composition^19,25^. Moreover when alien species invade a new area they are usually less parasitised than native or well established species as seen within a previous study from Minard *et al*. showing that the midgut microbiota of the French tiger mosquitoes had a lower richness than the Vietnamese one^20^. This suggests that adaptation to a different environment had a profound impact on the resident intestinal microbiota of this species, with potential impacts on its biology, and consequently on its vector competence. According to these findings, our hypothesis was that if the differences were driven by the adaptation to a new environment, we should expect French and Italian populations to have similar richness, given the comparable macro climate (temperate), type of breeding sites (urban settlements and human-made containers) and introduction period (end of nineties-beginning of millennium). Surprisingly, we found that the degree of microbial diversity in the midgut microbiota of Italian female *Ae*. *albopictus* was lower not only compared with Vietnamese, but also with French ones, suggesting the existence of other population-specific factors. These could be either a particular condition of the Italian population, or a larger and continuous higher rate of introduction of new individuals in France, probably due to international commercial trades. The higher variability shown by French and Vietnamese samples compared to Italian ones, can also be linked to specific microclimatic features of the sampling sites, such as vegetation and naturality, but also to the type of hosts available. We tried to minimize or exclude most of the confounding factors, which could affect variability among populations (previous blood meal, type of habitat and seasonality). All the sampled wild adult female mosquitoes analyzed in these studies were unfed; however, no further molecular tests were performed to exclude previous blood meals. Although *Aedes albopictus* colonizes edges of forests and breed in natural habitats in its country of origin (tree holes, bamboo stumps, and bromeliads), this species has adapted well to suburban and urban environments with larvae now breeding in artificial containers such as tires, cemetery urns, and water storage tanks. All the mosquito samples were collected in urban and suburban environments, with the exception of one out of four sites in Vietnam. The year of collection was different for the Italian (2015, this study) and the French and Vietnamese samples (2012^20^), but the season of collection was optimal for this species for all countries. To the best of our knowledge, there are no studies on the role of geographic location and year as factors affecting individual gut microbiota and we believe that such factors should be considered when comparing seasonality abundances or other ecological factors affecting population dynamic. In order to better assess the reasons of the differences among *Ae*. *albopictus* populations further comparisons with local mosquito species could be taken into account in the future.

The genus *Pseudomonas* dominated the microbiota of the Italian samples similarly to what reported in wild females of other species such as *Ae*. *aegypti* in Brazil^26^, *Culex pipiens* in Belgium^27^, and in *Anopheles stephensi* and *An. culicifacies* in southern Iran^28,29^. The interaction of this bacteria with pathogens transmitted by mosquitoes was observed in the case of *Anopheles* and *Plasmodium* with both positive and negative effects^30,31^. Moreover it has been reported that the presence of *Pseudomonas* helps in facing the oxidative stress resulting from the catabolism of blood meal^32^ which could cause a serious damage to insect cells. This bacteria has also been recently indicated as a good candidate to paratransgenesis since it grows in ordinary culture media and it is suitable for genetic transformation^27^.

The large core microbiota among the two *Aedes* species suggests a common environmental exposure in the breeding sites. In fact, the two mosquito species both share the same artificial habitats in urban settlements. Nonetheless, *Ae*. *albopictus* showed a higher richness and a different composition, as highlighted by α- and β-diversity. This could be partly explained by the fact that *Ae*. *albopictus* colonized the area about 15 years before *Ae*. *koreicus*.

Along with *Pseudomonadaceae*, other bacterial families were identified in our *Aedes* samples (like *Enterobacteriaceae*, *Acetobacteraceae*, *Rickettsiaceae* and *Moraxellaceae*). These were already found in different species of mosquitoes collected from geographically distant locations, supporting the hypothesis that many components of the midgut are cosmopolitan, well established as commensals, and have an important role in insect life cycle^33–38^. The role of *Enterobacteriaceae* is considered important in the digestion of blood in hematophagous *Diptera* and is frequently recovered in female mosquitoes of various species^38–41^. Some species of this family could also be used for a paratransgenic approach; for example *Enterobacter cloacae* has been found to block the development of *Plasmodium falciparum* in *An*. *gambiae* and sporogonic development of *P*. *vivax* in *An*. *albimanus*^42,43^ and to induce the expression of mosquito immune components in midgut of *An*. *stephensi*^44^. Other representatives from this family (genus *Serratia*) have a role in the suppression of the immune response of *Ae*. *aegypti*, therefore increasing its susceptibility to chikungunya and dengue viruses^45,46^.

A special attention should be focused on the contrasting presence of *Wolbachia* and members of the *Acetobacteraceae* family, most probably *Asaia* spp, in the two mosquito species. These two symbionts are the only bacteria known to be located in the reproductive organs of mosquito species, therefore vertically transmitted. The relationship between *Wolbachia* and mosquitoes is one of the most extensively studied among microbes harbored by insects^47–51^ as it is commonly found in mosquitoes midgut (with the notable exception of *Ae*. *aegypti*^52^), but also in somatic cells and gonads of *Orthoptera*^53^ and *Rhyncota*, such as bedbugs^54^. The most known effects of *Wolbachia* presence is the cytoplasmatic incompatibility between infected males and uninfected females, and its ability to inhibit the horizontal transmission or reduce the vector competence of some mosquito-borne pathogens like *Plasmodium* spp., dengue, yellow fever, West Nile fever and chikungunya viruses^52,55–62^. Once the vector potential of *Ae*. *koreicus* will be assessed, the lack of this endosymbiont could have important implications for the management of public health risk. Since the vectorial competence of *Ae*. *koreicus* for chikungunya virus was recently assessed by Ciocchetta *et al*.^63^ more studies are now necessary to better assess the consequences of the lack of this endosymbiont on the enhancement of its vectorial capacity.

*Asaia* spp. is a group of acetic acid bacteria that can be found in different niches like nectar-bearing flowers. Recently, this genus has been reported to be stably associated with different mosquito species, often being the dominant microorganism of the mosquito microbiota. *Asaia* has been widely reported in lab-reared^64^, but also in wild caught *Ae*. *albopictus* species^41^ and it seems to compete with *Wolbachia* in the ability to infect reproductive sites. Rossi *et al*. shown the infection of *Asaia* in the gut, but not in the reproductive organs of naturally infected *Ae*. *albopictus* and this was further confirmed by the absence of *Asaia* from the egg surface of this mosquito species^65^. Our findings reinforce the theory that mosquito-species naturally uninfected with *Wolbachia* (i.e. *An*. *gambiae* and *An*. *stephensi*^66^, *Ae*. *aegypti*^67^, *Ae*. *koreicus* (this study)) host *Asaia* spp. in various anatomical districts (midgut and ovary) and vice-versa, the occurrence of *Asaia* spp. in mosquitoes infected with *Wolbachia* is low. Most of the studies regarding the role of *Asaia* in mosquitoes, are related to several *Anopheles* species. In particular, it has been shown that *Asaia* spp. accelerates^68^ or has a beneficial role^69^ on larval development, with positive consequences on survivorship and competition with other mosquito species. In our study area, the two *Aedes* species overlap, therefore the presence of these bacteria needs to be further investigated. The consequences of microbiota composition in term of vector competence require more experimental studies since it has been previously assessed that the midgut bacteria play an important role in increasing or decreasing the vectorial capacity of the mosquito. Moreover it has been proven that vector-borne diseases can spread outside their endemic areas provided that the vector is present. An example are the chikungunya outbreaks in Italy in 2007^70^ and 2017^71^. Northern Italy is experiencing a regular introduction of infected chikungunya and dengue viruses human cases every year, while West Nile and Usutu viruses are endemic^72–74^. The presence of a species such as *Ae*. *koreicus* which has better chances to survive to colder temperatures^24^, its proved competence in transmitting pathogenic viruses such as chikungunya and the lacks of *Wolbachia* which potentially inhibits the circulation of these viruses, enhances the chances of spreading of exotic pathogens.

## Conclusions

In conclusion, the invasion of alien mosquito species into new areas challenges their gut microbiota in terms of reduction of richness and diversity, with possible consequences on the mosquito fitness and disease hazard. Local sampling conditions, though, could affect the gut microbiota composition and further research is necessary to investigate the effect of environmental factors on all developmental stages. With this study we provided an improvement of our knowledge of the highly diverse community of micro-organisms present in the midgut of two invasive mosquito species of public health concern.

## Methods

### Samples collection

Mosquito collections were performed in July and August 2015 in the Autonomous Province of Trento (Trentino-Alto Adige region, Italy), which is an area of recent invasion by the two studied species. This territory covers an area of 6 200 km^2^ with more than 70% lying above 1 000 m a.s.l. Only non-fed females were considered for the microbiota analysis. Thirty females belonging to the species *Ae*. *albopictus* and 30 to *Ae*. *koreicus* were included. Most of the specimens were collected by aspiration and CO2-baited BG sentinel traps, with the exception of 3 samples of *Ae*. *koreicus* which were captured by human landing technique. All the sampling sites were located in urban and suburban habitats. Mosquitoes were kept alive until the arrival in laboratory and then killed with a brief exposure to low temperature (−20°). Prior to dissection under stereomicroscope, specimens were identified to species level on the basis of morphological features^24,75^.

### Midgut isolation and DNA extraction

Before dissection, mosquitoes were surface sterilized in ethanol 70% for few minutes and rinsed with sterile water for molecular analysis. A sterile drop of PBS (100 µl) was used on the slide to facilitate the extraction of midgut. Only mosquitoes without visible traces of blood in the midgut were chosen for the study. Sterilized instruments were used for each dissection while the stereomicroscope was frequently cleaned with absolute alcohol. Midguts were put in sterile tubes and incubated overnight with ATL buffer and proteinase K at 56 °C with a gentle shaking (Thermo-shaker Grant Bio) prior processing. DNA extraction was performed using the Qiamp DNA Investigator kit (Qiagen) protocol “tissues” following the manufacturer’s protocol. Final elution volume was 25 µl (ATE buffer). DNA was quantified with fluorometer Qubit 2.0 (Invitrogen).

### Amplicon library preparation and sequencing

We sequenced genomic DNA of 10 *Ae*. *albopictus* samples targeting a domain of ~280-bp of the V5-V6 hypervariable region of 16 S rRNA gene using the specific primer 784 F (5′-AGGATTAGATACCCTGGTA-3′) and 1061 R (5′-CRRCACGAGCTGACGAC-3′)^76^ with overhang Illumina adapters. Genomic DNA of 30 *Ae*. *albopictus* and 30 *Ae*. *koreicus* samples were sequenced targeting a domain of ~460-bp of the V3-V4 hypervariable region of 16 S rRNA gene using the specific primer 341 F (5′ CCTACGGGNGGCWGCAG 3′) and 805Rmod (5′ GACTACNVGGGTWTCTAATCC 3′)^77^. PCR conditions, library preparation and sequencing followed the same protocol for all sequencing. Total genomic DNA was subjected to PCR amplification. All PCRs were conducted in 25 µl of volume and prepared under sterile conditions. Each PCR reaction contained 2.5 μL of 10 × Fast Start High Fidelity Reaction Buffer (Roche), 0.5 μL of 10 mM dNTP mix (Fermentas, UK), 1 μL of 10 μM forward and reverse primer, 0.25 μL of 5 U/μL Fast Start High Fidelity Enzyme blend (Roche), DNA (12 ng/μL) and sterile water to reach the volume. Reaction without template served as negative control. All PCR amplifications were carried out using a Veriti-96 Well Thermal Cycler (Applied Biosystem) and the following steps were set: melting step; 94 °C for 3 minutes (one cycle), annealing step; 94 °C for 15 seconds, 55 °C for 45 seconds, 72 °C for 1 minute and 10 seconds (35 cycles), extension step; 72 °C for 8 minutes (1 cycle). The PCR products were checked on 1.5% agarose gel and cleaned from free primers and primer dimer using the Agencourt AMPure XP system (Beckman Coulter, Brea, CA, USA) following the manufacturer’s protocol. Subsequently dual indices and Illumina sequencing adapters Nextera XT Index Primer (Illumina) were attached by 7 cycles PCR (16 S Metagenomic Sequencing Library Preparation, Illumina). The final libraries, after purification by the Agencourt AMPure XP system (Beckman), were analysed on a Typestation 2200 platform (Agilent Technologies, Santa Clara, CA, USA) and quantified using the Quant-IT PicoGreen dsDNA assay kit (Thermo Fisher Scientific) by the Synergy2 microplate reader (Biotek). Finally all the libraries were pooled in an equimolar way in a final amplicon library and quantified using the KAPA Library quantification kit by the real time qPCR LighCycler 480 (Roche). Barcoded library were sequenced on an lllumina® MiSeq (PE300) platform (MiSeq Control Software 2.0.5 and Real-Time Analysis software 1.16.18).

### Data analysis: *Aedes albopictus*, V5-V6 hypervariable region

Since the same region of the 16 S rRNA gene and the same primers of Minard and colleagues^20^ were used to sequence our 10 samples of *Ae*. *albopictus*, it was possible to analyze the sequence data from the two studies using the same protocols. Forty samples from Minard *et al*.^20^ were downloaded from the Sequence Read Archive (SRA, https://www.ncbi.nlm.nih.gov/sra) under the study ERP006543 (BioProject PRJEB6896). After paired-end alignment and primer trimming a total of 1 624 783 reads from Italy and 8 015 969 reads from France and Vietnam were quality filtered with an EE threshold of 0.5% and a minimum length of 250 bp. Reads with less than 60% similarity to the Greengenes database (May 2013 version, clustered at 85% identity) were removed using VSEARCH. Merging together all the remaining sequences we obtained 9 361 654 high quality reads. Reads were pre-processed using the MICCA pipeline (http://www.micca.org, version 1.6)^78^. After forward and reverse primer trimming, reads with an expected error rate (EE)^79^ higher than 0.5% and shorter than 400 bp were discarded. Reads with less than 60% similarity to the Greengenes database (May 2013 version, clustered at 85% identity)^80^, were removed using VSEARCH version 1.9.5^81^. 6 101 971 high quality reads were clustered de novo at 97% identity using the “de novo greedy” Operational Taxonomic Unit (OTU) picking method implemented in MICCA. The representative sequences were classified using the consensus classifier^82^ against the Greengenes database (May 2013 version) clustered at 97% identity. The sequences were aligned using the NAST^83^ multiple sequence aligner and a phylogenetic tree was inferred using the FastTree software available in the MICCA pipeline. Downstream analyses were performed in R environment using the phyloseq^84^, vegan^85^ and picante^86^ libraries. Midgut samples were rarefied (without replacement) (see Supplementary Fig. 3S) at 90 000 reads per sample. A randomization sampling test was performed in order to rule out any bias in the taxonomic classification^87^ (Supplementary Fig. 4S). The average proportion error was calculated as the relative difference between the relative abundance of each taxon in the whole library (i.e., with the maximum number of reads,9,361,654) and in the random sample (20 random samples for each sample depth). As expected, the consistency of the estimations of taxa abundances grows as the sample depth increases.

### Data analysis: Aedes albopictus and Aedes koreicus, V3-V4 hypervariable region

High Throughput Sequencing resulted in a total of 7 186 454 reads (after paired-end read alignment and merging). Pre-processing, filtering, clustering, taxonomic classification, multiple sequence alignment and phylogenetic tree inference was applied as for the V5-V6 samples. Samples were rarefied (without replacement) (see Supplementary Fig. 5S) at 45 000 reads per sample and one sample with 11 416 reads was removed. The core microbiota of all samples is composed by OTUs seen more than 10 times in at least 50% of the samples in each species. The species specific microbiota is composed by OTUs seen more than 10 times in at least 50% of the samples in one species and in less than 5% of samples in the other. Indicator species analysis was performed using the R package *indicspecies*^88^. P-values were FDR corrected.

### Bacterial community diversity

To quantify the bacterial richness in each mosquito species three different α-diversity estimators were used: the number of observed OTUs, the Chao 1 estimator and the Shannon entropy. To highlight the differences in the midgut microbiota of *Ae*. *albopictus* among countries and between *Ae*. *koreicus* and *Ae*. *albopictus*, we calculated the β-diversity using the unweighted and weighted Unifrac distances^89^ and the Bray-Curtis (BC) dissimilarity^90^.

Principal Coordinates Analysis (PCoA) was used to visualize differences between microbial communities in samples according to weighted and unweighted Unifrac measures and BC values. Statistical significance of the between-groups distances was assessed using PERMANOVA (Permutational Multivariate Analysis Of Variance Using Distance Matrices)^91^ (with 9 999 permutations), implemented in the *adonis* function in the *vegan* R-package. We defined as ‘core’ the set of OTUs with at least 10 reads in at least 50% of the samples from each species, and as ‘specific’ the set of OTUs with at least 10 reads in at least 50% of the samples from one species, but absent from the other (i.e. present in less than 5% of the samples). OTUs lesser than 0.05%, in at least 20% of the samples were removed.

## Electronic supplementary material

Supplementary Information

Dataset

## Data Availability

All data generated or analysed during this study are included in this published article (and its Supplementary Materials files).
