# Supplementary material for: Reduced diversity of gut microbiota in two Aedes mosquitoes species in areas of recent invasion
Source: Sci Rep. 2018 Oct 31;8:16091. doi: 10.1038/s41598-018-34640-z (PMC6208342; doi:10.1038/s41598-018-34640-z)
Supplement: Supplementary file 1 — Supplementary Information [file 41598_2018_34640_MOESM1_ESM.docx]

**Reduced diversity of gut microbiota in two *Aedes* mosquitoes species in areas of recent invasion.**

Fausta Rosso, Valentina Tagliapietra, Davide Albanese, Massimo Pindo, Frédéric Baldacchino, Daniele Arnoldi, Claudio Donati, Annapaola Rizzoli

**Supplementary Information files description:**

Supplementary Figures.docx: Supplementary figures 1S-2S

Supplementary Tables.xlxs S1-S14

Legends of the Supplementary Tables S1-S14

Table S1. Summary of relative abundances at Phylum level per country, median, first quartile (Q1), third quartile (Q3), mean and standard deviation (SD).

Table S2. Summary of relative abundances at Family level per country, median, first quartile (Q1), third quartile (Q3), mean and standard deviation (SD).

Table S3. Summary of relative abundances at Genus level per country, median, first quartile (Q1), third quartile (Q3), mean and standard deviation (SD).

Table S4. Alpha diversity indexes (observed number of OTUs, Chao1, ACE, Shannon, Simpson, inverse Simpson, Fisher) measured in each country. For each index median, first quartile (Q1), third quartile (Q3), mean and standard deviation (SD) are reported.

Table S5. Significantly different abundant OTUs (France vs. Italy, (P<0.01 DESeq’s Wald significance test, Benjamini & Hochberg FDR correction). log2FoldChange is the effect size estimate, pvalue is the p-value and padj is the FDR corrected p-value.

Table S6. Significantly different abundant OTUs (Vietnam vs. Italy, (P<0.01 DESeq’s Wald significance test, Benjamini & Hochberg FDR correction). log2FoldChange is the effect size estimate, pvalue is the p-value and padj is the FDR corrected p-value.

Table S7. Summary of relative abundances at Phylum level per species (Italian samples) country, median, first quartile (Q1), third quartile (Q3), mean and standard deviation (SD).

Table S8. Summary of relative abundances at Family level per species (Italian samples) country, median, first quartile (Q1), third quartile (Q3), mean and standard deviation (SD).

Table S9. Summary of relative abundances at Genus level per species (Italian samples) country, median, first quartile (Q1), third quartile (Q3), mean and standard deviation (SD).

Table S10. Alpha diversity indexes (observed number of OTUs, Chao1, ACE, Shannon, Simpson, inverse Simpson, Fisher) measured in each species (Italian samples). For each index median, first quartile (Q1), third quartile (Q3), mean and standard deviation (SD) are reported.

Table S11. Core OTUs in Italian samples. The core microbiome is defined by selecting OTUs seen more than 10 times in at least 50% of samples in each species.

Table S12. *Ae. albopictus* specific OTUs. The specific microbiome is defined by selecting OTUs seen more than 10 times in at least 50% of samples in *Ae. albopictus* species and not present in *Ae. koreicus* samples (< 5%).

Table S13. Indicator species analysis. Adjusted p-values are in the column p.value.adj (FDR correction).

Table S14. Significantly different abundant OTUs between *Ae. albopictus* and *Ae. koreicus* samples (Wilcoxon rank-sum test P<0.05, FDR corrected).

## Supplementary Figures


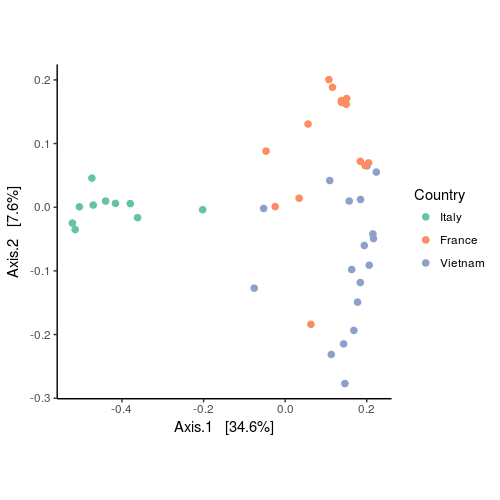


Figure 1S. Principal coordinates analyses (PCoA) using unweighted UniFrac distances.


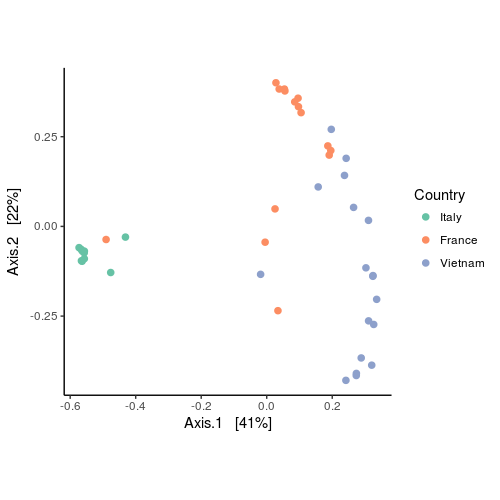


Figure 2S. Principal coordinates analyses (PCoA) using Bray-Curtis dissimilarities.
